# The pleiotropic roles of eIF5A in cellular life and its therapeutic potential in cancer

**DOI:** 10.1042/BST20221035

**Published:** 2022-12-13

**Authors:** Aristeidis Panagiotis Sfakianos, Rebecca Mallory Raven, Anne Elizabeth Willis

**Affiliations:** MRC Toxicology Unit, University of Cambridge, Gleeson Building, Tennis Court Rd, Cambridge, U.K.

**Keywords:** cancer, DHPS, DOHH, eIF5A, hypusination, translation

## Abstract

Protein synthesis is dysregulated in the majority of cancers and this process therefore provides a good therapeutic target. Many novel anti-cancer agents are directed to target the initiation stage of translation, however, translation elongation also holds great potential as a therapeutic target. The elongation factor eIF5A that assists the formation of peptidyl bonds during the elongation process is of considerable interest in this regard. Overexpression of eIF5A has been linked with the development of a variety of cancers and inhibitors of the molecule have been proposed for anti-cancer clinical applications. eIF5A is the only protein in the cell that contains the post-translational modification hypusine. Hypusination is a two-step enzymatic process catalysed by the Deoxyhypusine Synthase (DHPS) and Deoxyhypusine Hydroxylase (DOHH). In addition, eIF5A can be acetylated by p300/CBP-associated factor (PCAF) which leads to translocation of the protein to the nucleus and its deactivation. In addition to the nucleus, eIF5A has been found in the mitochondria and the endoplasmic reticulum (ER) with eIF5A localisation related to function from regulation of mitochondrial activity and apoptosis to maintenance of ER integrity and control of the unfolded protein response (UPR). Given the pleiotropic functions of eIF5A and by extension the hypusination enzymes, this system is being considered as a target for a range of cancers including multiple myeloma, B-Cell lymphoma, and neuroblastoma. In this review, we explore the role of eIF5A and discuss the therapeutic strategies that are currently developing both in the pre- and the clinical stage.

## Introduction

Aberrant control of mRNA translation is associated with the development of a range of diseases, particularly cancer [1]. The process of protein synthesis can be broken down into three stages initiation, elongation, and termination [2]. In terms of disease targeting most studies have focused on the initiation phase, which includes the assembly or the pre-initiation complex, scanning for the start codon and the formation of the 80S ribosome [2]. Extensive therapeutic strategies have been developed to target initiation, however targeting elongation also has great potential [3].

Elongation is, for the most part, controlled by eukaryotic elongation factors 1A1 and 2 (eEF1A1 and eEF1A2), GTPases that deliver cognate aa-tRNA to the A site of the ribosome and are released via GTP hydrolysis upon codon/anti-codon recognition [4,5]*,* and eukaryotic elongation factor 2 (eEF2), which mediates translocation of peptidyl-tRNA from A to P site of the ribosome. The activity of eEF2 is negatively regulated by phosphorylation via the kinase, eEF2K, which results in elongation stalling [5]. eIF5A is also required for elongation control, although its role in this process is less well understood [6]. eIF5A is a highly conserved protein from archaea (aIF5A) to yeast and human [6]. Bacteria poses EF-P which is the ortholog of eIF5A and similarly to eIF5A, also facilitates translation elongation [6]. eIF5A was initially thought to act as an initiation factor [7–9]. However, later studies describe its main function in promoting translation elongation [10–12]. EIF5A has two isoforms, eIF5A1 and eIF5A2 [13] of 157 amino acids [14,15], which share 84% amino acid sequence identity [16]. These proteins are differentially expressed [15]. eIF5A1 is the predominant isoform in most tissues, whereas eIF5A2 is highly expressed in brain, testis, and some malignancies [14,15].

**Figure 1.**
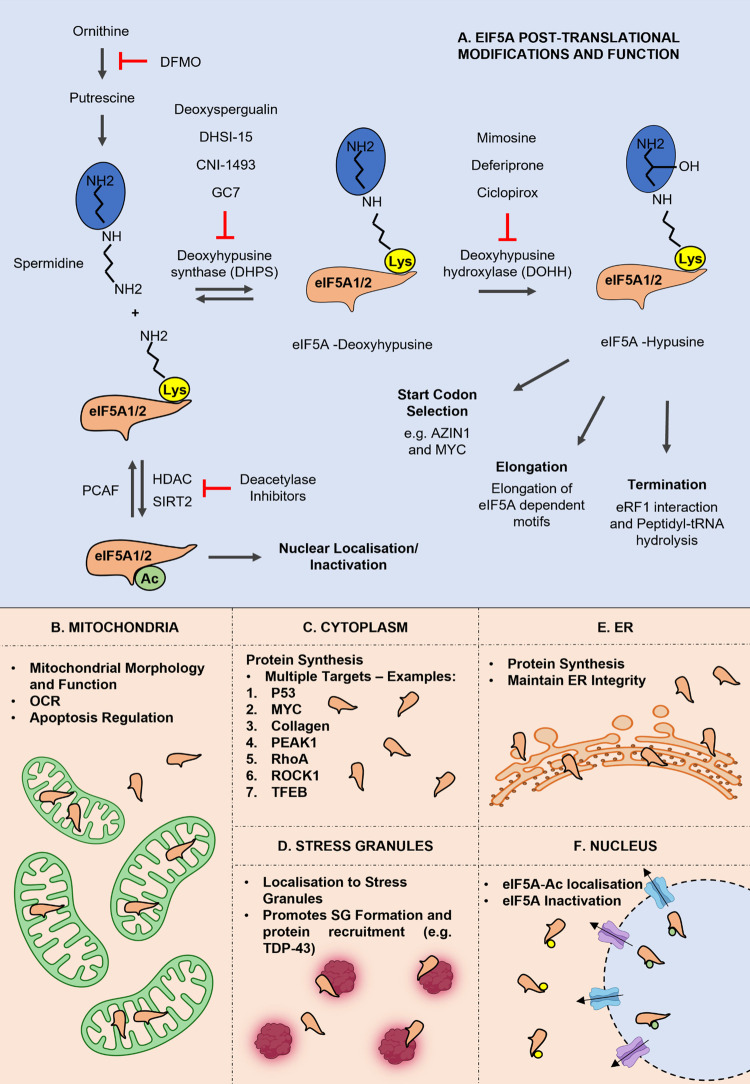
The pleiotropic functions of eIF5A. eIF5A isoforms 1 and 2 are hypusinated via a two-step enzymatic process that requires Spermidine as the donor of 4-aminobutyl moiety and the enzymes DHPS and DOHH. Hypusination activates the protein which takes part in start codon selection, translation elongation and termination. Non-hypusinated eIF5A can be acetylated by PCAF. Acetylated eIF5A translocated to the nucleus where it becomes inactivated. Deacetylases can reverse the modification and lead to translocation of the protein back to the cytoplasm. All enzymes that participate in eIF5A post-translational modifications can be deactivated via small molecule inhibitors. Apart from the cytoplasm and the nucleus eIF5A has been found to localise in mitochondria, the ER and stress granules under oxidative stress conditions. In each compartment eIF5A has a different functional role.

The two eIF5A isoforms are the only proteins in the cell that contain the post-translational modification hypusine [6,16]. Hypusination is carried out by a two-step enzymatic process involving deoxyhypusine synthase (DHPS) and deoxyhypusine hydroxylase (DOHH) and requires the polyamine Spermidine. In the first step DHPS catalyses the addition of the ε-aminobutyl moiety of Spermidine, which is the only known donor of the amino butyl group, on Lysine 51 of eIF5A for the reversible formation of the intermediate molecule eIF5A-deoxyhypusine (eIF5A-Dh). In the second step of hypusination, DOHH catalyses hydroxylation of the N6-(4-aminobutyl)-L-lysine intermediate of eIF5A and creates the final and active form of eIF5A, eIF5A-hypusine (eIF5A^H^). Hypusination of eIF5A activates the protein which assists in the progression of translation elongation (Figure 1A) [10,15,17,18].

A correlation between expression of eIF5A and cancer has been established and both eIF5A1 and eIF5A2 have been proposed to act as oncogenes [19,20]. Thus, it has been suggested that they have a role in driving hepatocellular carcinoma, gastric, colorectal, oesophageal, breast, ovarian, cervical, lung, bladder and prostate cancers [20–29]. In most of these cases eIF5A1 or 2 overexpression has been associated with increased cancer cell proliferation in cell models and poor survival prognosis in patients [20–29]. Although the mechanism(s) via which eIF5A functions in cancer development is unclear, recent data have revealed a pleiotropic mode of action for the protein in different types of tissues and organisms.

## eIF5A and mRNA sequence dependence

eIF5A is the ortholog of bacterial translation elongation factor EF-P [30]. In bacteria, EF-P is required to release elongation blocks in poly-proline motifs [30]. Similar behaviour has been observed for the yeast ortholog, eIF5A [11]. Initially, it was shown that in yeast a lack of eIF5A reduced the expression of a reporter gene containing poly-proline motifs [11]. Following studies demonstrated that eIF5A is important for resolving ribosome stalling in poly-Proline (PPP) or Proline–Proline–Glycine (PPG) stretches on endogenous yeast mRNAs [11,31]. Surprisingly, ribosome profiling in an eIF5A depleted yeast strain showed that eIF5A is not necessary only for elongation of poly-proline motifs, but possibly all mRNAs, suggesting a more global function for eIF5A in yeast elongation [12,32]. l Similarly, eIF5A appears to have a more generalised role in translation elongation in human cells [33]. In higher eukaryotes there is greater propensity of Pro–Pro–Pro or Pro–Pro–Gly triplets [34], such that in the human proteome 24% of proteins contain at least one Pro–Pro–Pro and 20% a Pro–Pro–Gly in their sequence. In *E. coli* only 5% of the proteins contain the inhibiting tripeptides and only 12% in yeast [34]. Although this takes on account the entire proteome and not the abundances of the individual proteins that also could be associated with translation efficiency and poly-proline-eIF5A dependencies, it is tempting to hypothesise that mammalian cells have evolved a more efficient translation machinery that does not rely upon eIF5A for elongation of most poly-proline stretches and suggests that the role of eIF5A is broader than originally thought. Indeed, a global quantitative proteomics approach revealed that eIF5A knock-down in HeLa cells was not linked with exclusive down-regulation of proteins containing poly-proline stretches [33]. It should be noted though that the dataset had small size of 972 protein targets that could represent the most abundant proteins in the cells and not the entire altered proteome [33]. Subsequently, knock-down of eIF5A in HeLa cells failed to reduce expression of Ribosomal Protein S6 Kinase 2 (S6K2) despite the presence of poly-proline motifs in the corresponding mRNAs [35]. On the contrary, eIF5A has been proposed to alleviate ribosomal stalling on an upstream conserved coding region (uCC) of AZIN1 by promoting elongation of a PPW motif that resides at the end of the uCC regulating the expression of the protein [36]. Moreover, translation of collagen proteins, which have multiple prolines containing motifs, is also eIF5A dependent with, chemical inhibition of eIF5A activity (with the drug GC7, see below) in mouse fibroblasts reducing expression of collagen proteins [37]. Finally, it has been shown that eIF5A and selectivity for poly-proline motifs in mammalian cells is also required for the regulation of MYC expression [21]. Simultaneous mutations of 5 stalling motifs on MYC mRNA rescued protein level reduction observed after knock-down of DHPS [21]. Mutations of individual poly-proline motifs did not affect the inhibition of expression observed in the absence of DHPS suggesting a role for the quantity and placement of these motifs in the protein sequence [21]. Taken together, these data suggest that although eIF5A has a broad role in elongation it likely also has a role in alleviating ribosomal stalling in tripeptides that contain consecutive prolines.

The role of eIF5A in the other two branches of translation, initiation, and termination, are less well understood. However, recent studies have revealed function for eIF5A in both processes [12,32,36,38]. Although a role for eIF5A in 80S formation has been dismissed, the elongation factor may regulate translation initiation in other ways. For example, in yeast loss of tif51a-1 leads to accumulation of ribosomes at the start and stop codons [32] and as mentioned above, in human cells, eIF5A controls canonical translation initiation by filtering ribosomal scanning in upstream coding conserved regions (uCCs) upstream AZIN1 [36]. In a similar manner eIF5A regulates start codon selection by supressing ribosomal pausing upstream canonical AUGs where proline dipeptides exist [38]. Interestingly, ribosome profiling data revealed that loss of eIF5A or DOHH enhanced upstream translation initiation of certain proteins including MYC [38].

eIF5A also plays an important role in translation termination [12,32]. Two independent studies have shown that depletion of eIF5A in yeast leads to accumulation of translating ribosomes in 3′ UTRs and stop codons. 3′ UTR accumulation results in a global defect of translation termination [12,32]. *In vitro* data suggest that addition of eIF5A^H^ to the termination reaction was able to promote peptidyl-tRNA hydrolysis mediated by eRF1 adding another level of regulation of eIF5A to termination [12]. Finally, recent structural analysis of eIF5A bound to the stalled ribosome has revealed that eIF5A can bind on the empty E-site that lacks tRNA suggesting that eIF5A is able to alleviate stalls via this route playing a major role not only in elongation but in every step of mRNA translation [39].

## eIF5A localisation and cellular function

Protein localisation defines protein function [40]. The predominant function of hypusinated eIF5A pertains to translation elongation while extending to further branches of translation regulation [11,12,32,38]. EIF5A has also been found in the nucleus, with acetylation of eIF5A by p300/CBP-associated factor (PCAF) linked with its translocation [41]. Treatment with HDAC inhibitors (TSA/NA) or the hypusination inhibitor GC7 increase acetylation of eIF5A and nuclear translocation [41]. Two different mechanisms have been proposed for eIF5A nuclear transfer out of the nucleus [42,43]. The first involves exportin 4 (Xpo4), which in complex with RanGTP interacts with eIF5A and facilitates its export from the nucleus [43]. The second suggests that eIF5A passively translocates to the nucleus with a role for Chromosomal Maintenance 1 (CRM1), in this process [42]. It has been proposed that CRM1 (or otherwise known Xpo1) in an exportin that leads to translocation of the protein from the nucleus, through the nuclear pore, to cytoplasm and subsequently to mitochondria where it induces apoptosis (Figure 1F) [42]. Two studies have suggested that an eIF5A1 isoform, variant A (eIF5A1VA), which results from an alternative start codon, has an additional N-terminal domain that acts as a mitochondrial targeting sequence (MTS) (Figure 1B) [44,45]. Down-regulation of eIF5A1VA leads to decrease in the cytoplasmic-expressed mitochondrial proteins ATP5A and SDHB and these data are similar to those observed for the canonical eIF5A1 variant B [44,46,47]. In addition, depletion of either eIF5A1VA or eIF5A1VB leads to decline of mitochondria fitness [44,46,47]. This is likely related to the fact that eIF5A is responsible for the expression of several mitochondrial enzymes including SDHA and B although the mitochondria located eIF5A1VA might have additional, currently unknown, functions in the organelle [44,46,47]. These data are consistent with studies which show that in macrophages, the polyamine synthesis pathway, which is important to produce spermidine and subsequent eIF5A hypusination, modulates oxidative phosphorylation (OXPHOS), maintains the Tricarboxylic Acid Cycle (TCA) and electron transport chain (ETC) integrity [46]. Moreover, increases in dietary spermidine were shown to rescue eIF5A hypusination loss and recover mitochondrial health in the aging brain of drosophila and mouse models [48,49]. In a mouse model for kidney ischemia GC7 reduced eIF5A hypusination, inhibited OXPHOS and silenced mitochondria causing a metabolic switch to anaerobic metabolism [50]. A recent study has demonstrated that supplementation with spermidine reversed the effects of eIF5A and hypusination depletion to the mitochondria of AML hepatic cells by preserving eIF5A^H^ levels [47]. It is noteworthy that overexpression of eIF5A1, although counterintuitive, has been linked to an increase in apoptosis via the intrinsic mitochondrial pathway in HT-29 and HeLa cells [51]. Overexpression of eIF5A1 was shown to result in up-regulation of p53 in HeLa cells, in agreement with studies that suggest that p53 translation requires eIF5A in HCT-116 and MCF7 cells treated with UV and Doxorubicin [51,52]. A major question arises: why does depletion of eIF5A have the same apoptotic effects as overexpression? One possible explanation for these data is that excess eIF5A can have a negative impact on mitochondrial function, due to accumulation of higher amounts of unhypusinated eIF5A [52]. Indeed, overexpression of the mutant eIF5A1 (K50A) that cannot be hypusinated induced activation of apoptosis by translocation of Bax to the mitochondria and release of cytochrome c, the same way as overexpression of the WT eIF5A [52]. Additionally, in H9c2 cardiomyocytes, increased eIF5A1 expression as a result of Doxorubicin treatments, resulted in dampening of mitochondrial fitness and induction of apoptosis [53]. Thus, modulation of eIF5A1 expression and hypusination are important for maintaining mitochondrial health ensuring cell survival.

eIF5A has also been observed in the ER (Figure 1E) [33,37,54,55]. One expected function of eIF5A in the ER is to promote protein synthesis [56]. However, additional functions have been proposed. In *S. cerevisiae* eIF5A has a role in the cotranslational translocation of proteins into the ER by binding to factors specific for this process [54]. Depletion of eIF5A in this model resulted in accumulation of unfolded proteins in the cytoplasm and induction of stress induced chaperones [54]. In agreement with this, depletion of eIF5A by using shRNAs against the protein leads to activation of the unfolded protein response (UPR) with an increase in phosphorylation and activation of PERK and increase in ATF4 and CHOP levels [33,57]. A global quantitative proteomics analysis revealed that depletion of eIF5A also leads to an increase in ER stress response proteins including protein chaperones such as HSPA5 and HSPA1B [33]. Moreover, additional studies have also shown that there is accumulation of Col1a1 in the ER following eIF5A knock down in mouse fibroblasts triggering the ER stress response [37]. Indeed, in this case BIP, ATF6 and CHOP mRNAs were up-regulated within 24 h following inactivation of eIF5A via GC7 indicating activation of UPR [37]. Taken together eIF5A appears to play multiple roles in the ER. It is important for maintaining general protein synthesis, correct protein folding and ER integrity by ensuring collagen expression. The different localisation points of eIF5A and compartmentalised functions are summarised (Figure 1).

## eIF5A and cellular signalling

eIF5A, especially in cancer, has been linked to the regulation of various signalling molecules. As discussed above, eIF5A can regulate the translation of MYC oncogene in colorectal cancer cell models via alleviating stalls in ribosome pausing sites either upstream the AUG or in the coding sequence [21]. Moreover, it has been shown that amplified MYCN in a neuroblastoma cell model could lead to increase in the transcription of DHPS suggesting a positive feedback loop regulation model for the hypusination pathway and MYC [58]

Another tumour associated protein that is regulated by eIF5A is the tumour suppressor protein p53 [59]. Overexpression of eIF5A in the kidney cell line COS-7 up-regulated p53 expression and its downstream effectors p21 and Bax [60]. In addition, stress induced p53 expression was suppressed in HCT-116 and MCF7 cells treated with GC7 [52]. In the same system ectopic expression of eIF5A also induced the expression of the protein [52]. Down-regulation of p53 also reduced the expression of apoptotic genes such as Bax and Bcl-2 inhibiting apoptosis following UV treatment of HCT-116 cells [52]. This is intriguing as in this, and multiple other cell lines, GC7 treatment alone was sufficient to reduce cell proliferation and induce cell death or senescence [21,52,58,61]. It is important to understand how eIF5A can help to alleviate elongation stalls under stress conditions for cancer treatments as often tumour cells grow under stressful environments [62–64]. In the lung adenocarcinoma cell line A549, hypusinated eIF5A was shown to be important for hypoxia-inducible factor 1-alpha (HIF-1A) protein induction under hypoxia [23]. Lack of eIF5A or its inhibition of it led to a reduction in tumour spheroid growth revealing a function of eIF5A, contradictory to the previous study [65]. In agreement with this, eIF5A was found to be important for the induction of cytoplasmic stress granules (SGs) [66,67]. Stress granules are cytoplasmic bodies formed by a process called liquid–liquid phase separation as a response to cellular stress that stalls translation initiation either by phosphorylation of eIF2α or by inhibition of eIF4A1 [68,69]. Knock-down of eIF5A1 or eIF5A2, significantly reduced the formation of SGs following arsenite treatments in RDG3 and U2OS cells [66]. A following study proposed that TDP-43, a SG protein, interacts with eIF5A allowing its colocalisation to SGs following arsenite treatment (Figure 1D) [67]. Reduction in hypusination inhibited binding of eIF5A to TDP-43 and accumulation of the latter to stress granules [67]. Taken together eIF5A could be a regulator of SGs formation and function and through this protect cancer cells from cellular stress [69].

Murine pancreatic intraepithelial neoplasia (PanIN) and human PDAC tissues have increased levels of hypusinated eIF5A [70]. In PDAC cells kRas activation leads to increased eIF5A expression [70] and this is directly linked to cell growth [70]. It has been proposed that eIF5A is able to control PDAC cell growth via the nonreceptor tyrosine kinase (PEAK1) expression regulation [70]. Indeed, overexpression of PEAK1 was able to rescue PDAC cell proliferation in the absence of eIF5A1 and eIF5A2 [70]. Finally, inhibition of eIF5A via GC7 and reduction in PEAK1 expression, increased sensitivity of PDAC cells to the chemotherapeutic drug Gemcitabine, suggesting that this could be a potential strategy for future cancer treatments [70]. Additional proteomic analysis in PDAC cells lacking eIF5A revealed a relationship between eIF5A and cytoskeleton regulatory proteins that are associated with cell migration and metastasis [71]. This association has been further confirmed in a physiological setting with eIF5A depletion to be responsible for inhibition of cell migration and metastasis [71]. It was proposed that eIF5A regulates cell spreading on Type I collagen [71]. Additionally, knock-down of eIF5A in PDAC cells reduced the expression of ROCK2 and RhoA, two proteins pivotal for cytoskeletal network regulation suggesting that eIF5A can regulate metastasis via a ROCK2/RhoA mechanism [71].

Another pathway that polyamines and eIF5A are associated with is autophagy [72]. In yeast supplementation with spermidine increased acetylation of histone H3 and increased expression of the autophagy proteins ATG7, ATG11 and ATG15 that promoted autophagy in multiple organisms and increased their longevity [72]. Similarly, GC7 was able to induce autophagy in a human fibrosarcoma cell line 2fTGH [73]. Dietary spermidine increased eIF5A hypusination and promoted autophagy in mouse primary B cells [74]. Hypusinated eIF5A was able to induce autophagy via increasing expression of TFEB by releasing stalls in poly-proline motifs [74]. Since dysregulated autophagy has been linked with several malignancies, polyamine synthesis pathway and eIF5A^H^ might provide anti-cancer therapeutic strategies.

## eIF5A and pharmacological targeting in cancer

There is a clear connection between eIF5A levels and activity in cancer [20–29] suggesting that eIF5A and the hypusination pathways are good targets for anti-cancer drug development. Indeed, Difluoromethylornithine (DFMO) that targets ornithine decarboxylase (ODC), which is responsible for spermidine synthesis and eIF5A hypusination, has already been approved in clinic for various purposes [75]. These include African trypanosomiasis, excessive facial hair growth in women and child neuroblastoma [75–77]. A compound that has been used widely in research to inhibit eIF5A is GC7 [78,79]. GC7 inhibits eIF5A hypusination via a block of DHPS resulting in cell growth stall in various cancers [21,61,80]. Although the specificity and bioavailability of GC7 make it an unsuitable candidate for clinical trials on its own it may be a useful tool when used in lower doses and in combination with other compounds such as DFMO [58,81]. Co-treatments of GC7 and DFMO induced apoptosis activation in a neuroblastoma cell model [58]. Since spermidine alone can rescue some of the effects of eIF5A inactivation it makes sense to block both hypusination and polyamine synthesis pathways for maximum therapeutic potential [47,49,58]. Another similar therapeutic strategy would be the combinatorial treatment of GC7 and diethylnorspermidine (DENSPM) which induces spermidine catabolism via activation of the spermidine catabolising enzyme SSAT and oxidation of spermidine [46]. Indeed, treatments with DENSPM increased oxygen consumption rates (OCR) which indicates OXPHOS activation while reducing production of several polyamine compounds [46]. It would be intriguing to further evaluate the effect of DENSPM and GC7 although not much work has been carried out to date.

GC7 has also been used in combination with common chemotherapeutics in preclinical studies increasing their therapeutic potential [70,82,83]. Co-treatments of GC7 or eIF5A siRNAs with imatinib induced increased cytotoxicity in a human leukaemia cell model [82]. Additionally, treatments with GC7 boosted chemosensitivity of oral squamous carcinoma cells treated with Doxorubicin [83]. Although it is yet to be tested with GC7, eIF5A knock down increased sensitivity of PDAC cells to the chemotherapeutic drug Gemcitabine suggesting that co-treatments of Gemcitabine with GC7 or DFMO may provide a therapeutic scheme for pancreatic cancer [70].

Other compounds that inhibit eIF5A hypusination have yet to be studied in detail [84–88]. These include the DHPS inhibitors Deoxyspergualin, CNI-1493 and DHSI-15 and DOHH inhibitors ciclopirox, deferiprone and mimosine [84–88]. Ciclopirox can inhibit cell growth in pancreatic, breast and other cancer cell lines, and has also been used in a Phase 1 clinical trial [24,89]. Deferiprone, is an iron chelator that has been approved for use by FDA for thalassemia similar to DFMO [90]. Since acetylation and nuclear localisation of eIF5A is tightly linked to its inactivation, lysine deacetylase inhibitors may be an interesting therapeutic approach for cancer [41]. An attractive tactic to target eIF5A is the use of an SNS01-T nanoparticle which encloses both an inactive mutant version of eIF5A (eIF5A-K50R) and siRNAs against the protein [91]. Use of this nanoparticle reduced growth of a B-cell cancer model and it has been through early-stage clinical trials as a treatment for multiple myeloma and B-cell lymphoma [91]. A final perspective for anti-cancer treatment is the use or modulation of miRNAs [92,93]. eIF5A2 regulates miR33-b expression in myelotic Leukemia cell line (MCL-1) which in turn regulates sensitivity to Daunorubicin [93]. Additionally, inhibition of DOHH via mimosine is found to act in synergy with miR-331-3p and miR-642-5p that are targeting DOHH mRNA to inhibit cell growth in a prostate cancer cell model [92].

## Perspectives

- mRNA translation and protein synthesis are one of the most important pillars of cellular function. Dysregulation of protein synthesis has been linked to many diseases including cancer. A better understanding of protein synthesis regulation could pave the way for novel, more specific and effective therapeutic strategies for cancer and other diseases.
- eIF5A was originally thought to be an initiation factor. later studies revealed that eIF5A plays a major role in elongation and more specifically the formation of peptide bonds between prolines in poly-proline stretches preventing ribosomal stalling. On the evolution of this idea eIF5A is now considered playing a role not only in poly-proline stretches but translation elongation in general. However, depletion of eIF5A has been linked to reduction in protein levels of specific protein groups such as mitochondrial proteins and proteins associated with autophagy hinting to possible future directions for therapeutic strategies involving eIF5A function.
- There are plenty of outstanding questions about eIF5A function. Despite many papers discussing the significance of eIF5A in translation elongation the exact protein targets regulated by eIF5A have not been yet clarified. It is not clear whether eIF5A is important for relieving ribosomal stalling in poly-proline stretches in human cells and whether this is linked to specific protein synthesis and phenotypes. Additionally, eIF5A has been suggested to play several roles in translation that are not bound in the elongation stage but transcend to both initiation and termination, thus more experiments are required to completely unravel the role of this small but pleiotropic protein. Finally, given the unique position of eIF5A as the only protein with the post-translational modification hypusine many new hypusination inhibitors will be developed and new therapeutic strategies focusing on that will be designed as it has the potential to be a highly specific target for anti-cancer treatment.

## Open Access

Open access for this article was enabled by the participation of University of Cambridge in an all-inclusive *Read & Publish* agreement with Portland Press and the Biochemical Society under a transformative agreement with JISC.

## Abbreviations

ER: endoplasmic reticulum
ETC: electron transport chain
MTS: mitochondrial targeting sequence
OCR: oxygen consumption rate
OXPHOS: oxidative phosphorylation
SGs: stress granules
TCA: tricarboxic acid cycle
UPR: unfolded protein response
